# Are we ready to use anti-amyloid therapy in Alzheimer's disease?

**DOI:** 10.1590/0004-282X-ANP-2022-S117

**Published:** 2022-08-12

**Authors:** Sonia Maria Dozzi Brucki, Karolina Gouveia César-Freitas, Raphael Ribeiro Spera, Conrado Regis Borges, Jerusa Smid

**Affiliations:** 1 Universidade de São Paulo, Departamento de Neurologia, Unidade de Neurologia Cognitiva e Comportamental, São Paulo SP, Brazil.; 2 Hospital Israelita Albert Einstein, Grupo Médico-Assistencial de Memória e Cognição, São Paulo SP, Brazil.; 3 Instituto de Infectologia Emílio Ribas, São Paulo SP, Brazil.

**Keywords:** Alzheimer Disease, Biomarkers, Dementia, Doença de Alzheimer, Biomarcadores, Demência

## Abstract

Alzheimer’s disease (AD) is the most common neurodegenerative disease. Biomarkers have demonstrated that AD pathology exists over the disease continuum from a stage preceding symptoms over 15-25 years to the progressively more impaired symptomatic states, mild cognitive impairment (MCI), and dementia. Biomarkers include: amyloid (Aß), phosphorylated tau, and neurodegeneration. The plasma assays for Aß and tau show great promise for clinical and research use. This review has aimed not only to present the ATN diagnostic classification and the preclinical AD concepts in addressing some possibilities of cognitive assessment instruments, but also to briefly summarize the main anti-amyloid monoclonal antibodies studied in clinical trials. In addition, this paper presents a critical analysis by experts in cognitive neurology while addressing the question as to whether we are prepared for the anti-amyloid therapy era or not.

## RESUMO

A doença de Alzheimer (DA) é a doença neurodegenerativa mais comum. Os biomarcadores demonstraram que a patologia da DA existe ao longo do continuum da doença, desde um estágio anterior à sintomatologia, ao longo de 15 a 25 anos, até os estados sintomáticos progressivamente mais prejudicados, comprometimento cognitivo leve (CCL) e demência. Eles subdividem-se em três categorias: amiloide (Aß), tau fosforilada e neurodegeneração. A dosagem de Aß e tau plasmáticos mostra uma grande promessa para uso clínico e de pesquisa. Esta revisão teve como objetivo apresentar a classificação diagnóstica da ATN, os conceitos pré-clínicos da DA e abordar algumas possibilidades de instrumentos de avaliação cognitiva, mas principalmente resumir brevemente os principais anticorpos monoclonais anti-amiloide estudados em ensaios clínicos. Além disso, este artigo apresenta uma análise crítica de especialistas em neurologia cognitiva: estamos ou não preparados para a era da terapia anti-amiloide?

## INTRODUCTION

Answering the title question is very difficult without having some crucial knowledge about pathophysiology and well-defined terms and ideas. Therefore, the main aspects of disease-modifying drugs in the treatment of Alzheimer’s disease (AD), the most common and one of the best-characterized disorders among neurodegenerative diseases, are going to be addressed in what follows. 

## PATHOPHYSIOLOGY, AMYLOID CASCADE THEORY, AND ATN CLASSIFICATION

Classically, the entorhinal cortex of the medial temporal lobe, an essential pathway through information input to the hippocampus, is affected in early disease stages. Other regions involved that are involved early on in the disease process include: the *locus coeruleus*, dorsal raphe nucleus, posterior cingulate, precuneus, medial and lateral parietal, and lateral frontal cortices. AD neuropathological hallmarks have remained the same over the last 100 years since the initial description by Lois Alzheimer: (a) extracellular neuritic plaques (NP) - composed mainly of beta-amyloid peptide (βA42) deposited primarily in the parenchyma and in blood vessels and (b) neurofibrillary tangles (NFT) - intracellular cytoplasmic aggregates of the hyperphosphorylated tau protein (pTau)^1^. NP and NFT can be detected with silver stains, such as Bielschowsky or Gallyas, or by immunohistochemistry using antibodies against βA or Tau^1^.

Research criteria for the pathological diagnosis of AD involve the description of NP and NFT^2^. The Consortium to Establish a Registry for Alzheimer’s disease (CERAD) used semi-quantitative NP frequency adjusted to age, and Braak & Braak defined six stages according to the presence of NFT in prone regions of the brain. The National Institute of Aging (NIA) and the Alzheimer’s Association (AA) later proposed modifications. The 2012 NIA-AA is one of the most commonly used criteria, applies the “ABC score” that includes the Thal Aβ phase in classification^3^. Despite the local incongruence of overlap between NP and NFT, the best pathophysiology theory includes the “amyloid cascade theory”^4^, characterized by (i) formation, overproduction, and accumulation of βA42 derived from amyloid precursor protein (APP) leading to neuronal injury, formation of oligomers, protofibrils, and plaques after the two steps of cleavage of AAP by β and γ-secretase. The process later (ii) evolves with secondary tauopathy and NFT formation, and further. advances with microtubule dysfunction, leading to synaptic loss and retrograde degeneration, neuronal dysfunction, and, finally, brain atrophy. In summary, the hypothesis is that β-amyloidosis enables the spread of tauopathy, which is associated with neurodegeneration, the immediate cause of clinical symptoms. There are often vascular changes in small vessels and capillaries, with or without parenchyma alterations denoted by cerebral amyloid angiopathy (CAA). The hypothesis is that Aβ exits the brain via perivascular pathways, and that reduced perivascular clearance (rather than overproduction of Aβ) is a shared pathogenic mechanism in CAA and AD^5^.

Since 2016, the “A/T/N” classification system has been used in cognitive aging and dementia research^6^. This biomarker classification makes no assumptions as to temporal order of biomarkers or amyloid cascade and is independent of clinical and cognitive status. It is a binary system that evaluates the presence of neurodegeneration, βA, and tau protein. The “A” class refers to presence of a β-amyloid biomarker (high ligand retention on amyloid PET or low CSF Aβ42); the “T” refers to presence of a tau biomarker (elevation of CSF pTau, or high ligand retention tau PET); and, finally, “N” refers to biomarkers of neurodegeneration or neuronal injury (hypometabolism on [18F]-fluorodeoxyglucose-PET, atrophy on structural MRI in regions characteristic of AD or high CSF total tau). Each biomarker is categorized as positive or negative. An individual score might appear as A+/T+/N-, or A+/T-/N-, etc. (Table 1). If a biomarker class is unavailable, it would be categorized as “u.” Later, in 2018, the A/T/N classification was combined with the cognitive stage (cognitively unimpaired, mild cognitive impairment, or dementia)^7^.

**Table 1.** Biomarker profiles and categories.

AT(N) profiles	Biobiomarker category		
A-T-(N)-	Normal AD biomarkers	AlzhAlzheimer’s continuum
A+T-(N)-	AlzhAlzheimer’s pathologic change	
A+T+(N)-	AlzhAlzheimer’s disease	
A+T+(N)+	AlzhAlzheimer’s disease	
A+T-(N)+	AlzhAlzheimer’s and concomitant suspected non-non-Alzheimer’s pathologic change	
A-T+(N)-	Nonnon-AD pathologic change*	
A-T-(N)+	Nonnon-AD pathologic change*	
A-T+(N)+	Nonnon-AD pathologic change*			

*suspected non-Alzheimer’s pathophysiology or SNAP.

## PRECLINICAL AD DIAGNOSTIC CRITERIA

In 2016, the National Institute on Aging (NIA) and the Alzheimer’s Association (AA) set up a panel to develop a research framework conceptualizing AD primarily as a clinicopathological entity, since pathological changes can occur over 15-25 years without any symptoms^1^,^8^. Therefore, presence of biomarkers has demonstrated that AD pathology exists over the continuum of the disease from a stage preceding symptomatology (the “preclinical state”), which includes subjective cognitive decline (SCD), followed by the progressively impaired symptomatic states of mild cognitive impairment (MCI) and dementia.8 While the ATN system was adopted in research, the panel described the six-stage clinical staging of AD based on the clinical nosology (Table 2).

**Table 2.** Six-stage clinical staging of Alzheimer’s disease based on National Institute on Aging and Alzheimer’s Association.

Stage	Clinical characteristics	Syndromic stage
1	Normal cognitive performance, No reported cognitive decline or new onset of neurobehavioral symptoms	Cognitively unimpaired
2	Normal cognitive performance, Subjective cognitive decline, or Documented evidence of decline, or Newly acquired neurobehavioral symptoms	Cognitively unimpaired or SCD
3	Performance in impaired range, and Cognitive decline from baseline in any domain, and ADL independent, but may be less efficient	MCI
4	Mild dementia: Substantial cognitive impairment affecting several domains, and Clearly evident functional impact on daily life, and No longer fully independent	Dementia
5	Moderate dementia: Progressive cognitive impairment or neurobehavioral changes No longer independent and requires frequent assistance with ADL	Dementia
6	Severe dementia: Progressive cognitive impairment or neurobehavioral changes, Clinical interview may not be possible, Complete dependency due to severe functional impact on ADL with impairment in basic activities, including basic self-care.	Dementia

ADL: activities of daily living; SCD: subjective cognitive decline; MCI: mild cognitive impairment.

Irrespective of clinical symptoms, the term “Alzheimer’s disease” refers to an aggregate of neuropathologic changes andis thus defined *in vivo* by biomarkers or by postmortem examination. A minimum amount of amyloid plaques (composed of Aβ42 peptide) is necessary for the diagnosis of AD pathology^7^. Nevertheless, in order to fulfill AD criteria at any stage at least two biomarkers need to be identified ^7^ ^,^ ^8^. Currently, the interest in stage 1 diagnosis is reserved for research and clinical trials. On the other hand, multi-domain amnestic dementia phenotype is the most common presentation at stages 4-6, albeit not specific as it can occur in other diseases other than AD^7^. Nonamnestic clinical presentations such as language, visuospatial, and executive disorders, may also occur in AD, especially in younger individuals.

### Subjective cognitive decline

SCD is defined as self-perceived cognitive decline, with normal performance within the expected range on objective cognitive tests and no functional impairment on activities of daily life^9^. The decline may involve any cognitive domain(s), not exclusively memory, and may be corroborated by the informant, although this is not required^7^. In the context of predementia diagnosis, SCD is even more challenging because it may be caused by causes other than neurodegenerative diseases, such as AD ^10^. However, SCD has been recognized as the earliest symptomatic manifestation of AD in most research works^10^.

### Mild cognitive impairment

MCI is characterized by (1) cognitive complaint reported by the patient and/or informant; (2) report of cognitive decline over the previous year; (3) performance in the impaired/abnormal range on objective cognitive tests (memory and/or other domains), compared to normal adults of the same age and educational level; (4) preserved general cognitive functioning; (5) absence of dementia^11^ ^,^ ^12^.

## NEUROLOGICAL ASSESSMENT

Many cognitive tests are available for a brief assessment of the main cognitive domains. The Mini-Mental State Examination (MMSE) the most used evaluation tool worldwide, covers temporal and spatial orientation, memory, attention, calculations, language, and construction skills^13^. The MMSE can be used for screening, diagnosis, and progression assessment. It was adapted and standardized for the Brazilian population according to educational level^14^.

Another more recently commonly used test is the Montreal Cognitive Assessment (MoCA) which can be used in patients with MCI or mild dementia, particularly those with higher educational levels (>12 years)^15^. Consequently, using MoCA in a population with heterogeneous educational background, such as that of Brazil, may be limited^16^. In order to avoid this difficulty, the Brief Cognitive Screening Battery (BCSB) could be used to detect cognitive impairment in patients with both low and high education levels ^17^ ^,^ ^18^. This test consists of naming and encoding ten pictures and recalling them five minutes later, after an intervening activity consisting of semantic verbal fluency (animals/1 min) and the clock drawing test^19^.

Finally, a formal neuropsychological evaluation is required to assess other specific cognitive domains, such as language, praxis, visuospatial skills, episodic memory, attention, and executive functions, when usual cognitive screening tests are not sensitive enough to detect cognitive impairment, especially in MCI individuals, or when diagnostic uncertainty remains.

## BIOMARKERS USED FOR THE DIAGNOSIS OF ALZHEIMER'S DISEASE

Biomarkers for AD diagnoses are divided into three categories: Amyloid (Aß), Tau, and Neurodegeneration^7^ ^,^ ^19^.

### Biomarkers for Aß pathology

Amyloid-beta (Aß) is derived from the amyloid precursor protein (APP), a transmembrane protein concentrated in neuronal synapses. APP is created initially by a ßAPP cleaving enzyme 1 (BACE1) and then by gamma-secretase, yielding Aß peptide, composed of 37 to 43 amino acids. Isoforms with 42 and 43 amino acids are the most prone to aggregate in the extracellular plaques found in AD patients^20^. The diagnostic methods are positron emission tomography (PET) with Aß tracers and cerebral spinal fluid or serum amyloid assays.

*5.1.1 Amyloid PET*

PET is a nuclear medicine method widely used in Alzheimer's disease research. Amyloid tracers can detect insoluble Aß fibrils in plaques with high accuracy, using post-mortem pathological findings as gold standard^20^. Conversely, a negative Aß-PET scan allows ruling out the diagnosis of AD.

 Amyloid PET allows detection of amyloid accumulation early in the pathological AD process, about 20 years before dementia onset^19^ ^,^ ^20^. Initially, the most affected structures are the medial frontal and medial parietal regions, and, as the disease progresses, amyloid deposition spreads to the entire cerebral cortex. Therefore, presence of Aß plaques in cognitively normal people or people with mild cognitive impairment is associated with an increased risk of developing dementia.

 In Brazil the most commonly used PET tracer is the carbon 11 based Pittsburgh compound B ((C^11^) PiB). Major limitation is the rapid decay rate (about 15 minutes), requiring an in-site cyclotron. Fluor 18 based tracers have a longer half-life (about 180 minutes), facilitating logistics and allowing the exam to be performed far away from the manufacturing site. The most commonly used ^18^F-based tracers are (F^18^) florbetapir, (F^18^) florbetaben and (F^18^) flutemetamol^20^.

 Aß-PET scans have limitations: first, older adults often show Aß plaques, even though they may be cognitively normal. Second, location of amyloid plaques does not coincide with the clinical course of AD, preventing Aß-based methods to assess disease severity and prognosis^20^.

*5.1.2 Amyloid in CSF*

Aß can be accurately measured in the cerebrospinal fluid (CSF). Patients with AD showed about 50% reductions in CSF Aß42 concentrations. The Aß42/Aß40 ratio is also reduced, with better accuracy than isolated Aß42 sampling. In addition, a reduced Aß42 or Aß42/Aß40 ratio in the CSF of MCI patients predicts development of dementia with high accuracy^19^ ^,^ ^20^.

 Aß CSF measuring is an invasive method, still unavailable in several locations. Moreover, the method is susceptible to several pre- and post-analytical variables. Fortunately, standardized CSF collection and analysis protocols as well as development of new techniques have been published.

*5.1.3 Serum amyloid*

 Due to the high cost of Aß-PET and concerns with CSF collection, there has been an incentive to develop more accessible diagnostic methods, such as serum amyloid analysis. Until 2016, attempts to detect amyloid in serum were unsuccessful due to the very low concentrations of these peptides in the serum. With the development of hypersensitive methods (such as single-molecule array and immunoprecipitation mass spectrometry), Aß42 and Aß42/Aß40 rates in serum have become feasible^20^ ^,^ ^21^. However, the decrease in Aß42 in serum is much lower (10-20%) than in the CSF of patients with positive amyloid PETs (40-60%)^20^. Production of Aß at sites other than the central nervous system can explain this discrepancy. An alternative to address this matter is to use serum Aß in combination with other serum tests.

### Tau protein biomarkers

Tau Protein is an intracellular component that brings together and stabilizes microtubules. Its gene, MAPT, can be transcribed into six isoforms: three with three repeats, and three with four repeats. Tauopathy encompasses both three and four repeats' isoforms in AD patients^20^. The tau protein can be modified in more than 70 post-translational modification sites, including more than 40 phosphorylation sites^21^. In AD, hyperphosphorylation leads to formation of neurofibrillary tangles [DIdM1] within neurons and, consequently, neurodegeneration.

*5.2.1 Tau-PET*

The most widely used tau tracers are (F^18^) flortaucipir, (F^18^) MK6240, and (F^18^) RO948. These tracers show high sensitivity and specificity for the diagnosis of AD, even compared to other tauopathies^20^. Furthermore, in vivo tau tracer's regional uptake corresponds to tau pathology in post-mortem studies.

 In the early stages of typical AD, tracer accumulation begins in the entorhinal cortex, spreading to nearby areas, such as the hippocampus and subiculum. Therefore, progression of tau ligands in the cerebral cortex is similar to pathological tau deposition and neurodegeneration progression in AD described by Braak^20^ ^,^ ^22^. Because of this, the pattern of tau ligand deposition in the cerebral cortex is closely related to AD clinical phenotype. For example, typical amnestic symptoms are associated with symmetrical temporoparietal tau deposition, logopenic aphasia to asymmetric left temporal tau, and visuospatial impairment to posterior parietal tau^20^. 

 Tau-PET can be used both as a diagnostic biomarker for AD, and as a dementia risk predictor in patients with MCI or without cognitive impairment. In addition, Tau-PET is under study as a possible prognosis indicator^20^.

*5.2.2 CSF Phosphorylated tau*

 Total tau (t-tau) and phosphorylated tau (p-tau) have different implications in AD diagnosis. Total tau protein is not specific to Alzheimer's disease, as it is elevated in several neurodegenerative disorders. However, it is an excellent marker of neurodegeneration. In order to investigate AD tau pathology, one must analyze the phosphorylated tau protein. Phosphorylation of tau protein can occur at several sites. The two phosphorylation sites with the highest diagnostic accuracy are threonine 181 (P-tau-181) and 217 (P-tau-217). P-tau-181 and P-tau-217 are selectively increased in AD, but not in other neurodegenerative diseases. Moreover, they are associated with a higher risk of conversion of cognitively unimpaired or MCI individual to AD dementia^20^ ^,^ ^21^.

*5.2.3 Serum phosphorylated tau*

Ultrasensitive diagnostic methods have allowed the analysis of p-tau in serum with high sensitivity and specificity. In addition, serum P-tau-217 was equivalent to other methods used for predicting dementia risk in preclinical and prodromal AD cases^20^. Therefore, although still in the validation phase, P-tau217 detection in serum is a promising tool for use in clinical trials as a diagnostic method for preclinical AD. It could guide initiation of disease-modifying treatments, and monitoring treatment response. Additionally, it could help predicting prognosis and rate of disease progression^20^.

### Neurodegeneration biomarkers

The main biomarkers of neurodegeneration are magnetic resonance imaging (MRI), PET with fluorodeoxyglucose (FDG-PET), and total tau protein in CSF. In addition, newer studies have recently highlighted the measurement neurofilament light chain (NfL) in the serum and cerebrospinal fluid.

 MRI is essential for the initial diagnosis of dementia, allowing ruling out vascular dementia and causes of reversible dementias. In addition, the atrophy pattern can help narrow down diagnostic hypotheses in different neurodegenerative dementias^23^.

 In AD dementia, atrophy of mesial temporal structures predominates in the early stages. As the disease progresses, atrophy spreads throughout the cortex. However, observation of atrophy on MRI is neither sensitive nor specific for AD diagnosis. On the other hand, it helps monitor disease course. Sequential tests on the same patient show gradual accentuation of atrophy, especially in the mesial temporal region. The most accurate methods for such comparison are hippocampal volumetry, and analysis of cortical thickness^23^.

 FDG-PET detects metabolic activity in several cortical areas by capturing the signal emitted by a radioactive glucose molecule. In Alzheimer's disease, glucose uptake is reduced in the posterior cingulate, precuneus, temporoparietal cortex, and medial temporal cortex^23^. The main strength of FDG-PET over brain MRI is that hypometabolism occurs earlier than atrophy. While atrophy is usually seen only in AD dementia, metabolic impairment appears earlier in the prodromal stages of AD. In addition, FDG-PET has greater accuracy in the differential diagnosis between AD and other dementias^23^.

 NfL is an axonal scaffolding protein. When neuronal damage is present, an elevation of NfL occurs in both CSF and serum. NfL has a well-established use in various neurological diseases, such as Parkinson’s plus diseases, amyotrophic lateral sclerosis, and frontotemporal dementia. Recently, it has also been studied in patients with AD^21^ ^,^ ^23^. This biomarker indicates neurodegeneration, and it assists in estimating the rate of disease progression, prognosis, severity, and can enable monitoring response to disease-modifying therapies. NfL however, are not specific to Alzheimer's disease and does not help in the differential diagnosis with other causes of dementia^21^.

## ANTI AMYLOID THERAPY

Prevention of cognitive decline in AD is of major importance. The disease has a considerable cost, with an estimate of nearly 1 trillion USD around the globe^24^. Emerging AD-modifying therapy is a possibility towards hoping for fewer patients with the disease. 

 There are many challenges related to using this kind of therapeutics. The first challenge is an accurate and early diagnosis; before the stage of dementia (SCD or MCI), general practitioners must be trained to make a diagnosis and refer the patient to a patient-centered dementia care to perform biomarkers (PET scan, fluid-based or CSF-based) and to identify which patients are suited for modifying pharmacological interventions. Another drawback in the treatment is the necessary infrastructure (MRI, PET, and biomarkers facilities). Interventions should be appropriate, affordable, and accessible^25^.

 Amyloid therapy until now has not shown positive and robust results. It is not clear if removing amyloid will stop the processes involved in the physiopathology of AD^26^.

 The first approval of a disease-modifying therapy was in June, 2021 by the U.S. Food and Drug Administration (FDA), with considerable controversy about results shown by two clinical trials with aducanumab. We shall discuss this substance and others in the pipeline. 

### Aducanumab

Aducanumab is an amyloid beta-directed monoclonal antibody against Aß aggregates. The approval of this drug was based on the results of two phase-3 clinical trials (EMERGE and ENGAGE), which have included MCI and mild AD patients. The patients were followed for 78 weeks. The main inclusion criteria were age between 50 and 85 years, CDR 0.5 to 1, MMSE 24 to 30 points, and positive amyloid-PET. The primary outcome was CDR-SB (Clinical rating Scale-Sum of Boxes), and secondary outcomes were MMSE (cognition), ADAS-Cog-13 (cognition), and ADCS-ADL-MCI (instrumental activities of daily living). Biomarkers were also used for secondary outcome measurements (amyloid-PET, tau-PET, and CSF biomarkers)^27^.

 Although the two studies were identical, one was positive (EMERGE), with significant differences compared to placebo in CDR-SB ADAS-Cog-13. The difference in the placebo's primary outcome (CDR-SB) was 22%, slowing the progression in 78 weeks. The ADAS-Cog showed a 27% drug-placebo difference, the ADCS-ADL MCI demonstrated a 40% drug-placebo difference, and the MMSE an 18% difference^27^ ^,^ ^28^.

 The amyloid retention in PET was the most substantial effect in the active treatment arm. Another significant effect was the lowering of CSF p-tau^29^.

The patients in the EMERGE study were under a longer high-dose regimen. Doses ranged from 3 mg/kg to 10 mg/kg in patients divided into ApoE E4 carriers and non-carriers^27^.

 Two adverse events (AE) were relatively common, the Amyloid-Related Imaging Abnormalities, ARIA-E (edema), and ARIA-H (hemorrhage), present in 30.7 to 41.2% of patients, and more frequently seen in ApoE E4 carriers and in patients who received higher doses. AE presented as headache, dizziness, nausea, visual disturbances, with spontaneous resolution in four to 16 weeks^27^ ^,^ ^29^.

Since its approval, use recommendations have been established and prescription of aducanumab should be restricted in patients with mild AD or MCI, no clinically significant systemic illness, and no unstable psychiatric condition^30^. Another aspect is the monthly infusion and MRI at baseline and before the fifth, seventh, and 12^th^ infusions^31^.

 Considering “a real world” treatment scenario, the proportion of eligible patients would be low (less than 1%) as shown in an Italian outpatient dementia clinic^32^. Ninety-two percent of Medicare beneficiaries would be excluded from clinical trials with aducanumab^33^. 

 A negative aspect is the cost of this medication. The initial cost was US$56,000/year; after some months, Biogen reduced cost to US$28,000. The Institute for Clinical and Economic Review report calculated the cost-effectiveness threshold prices ranged from an annual price of US$2,560 to US$8,290^34^.

### Lecanemab

Lecanemab (BAN2401) selectively binds to large, soluble Aß protofibrils and oligomers and insoluble fibrils. It is a humanized IgG1 version of mAb158. The phase IIb study enrolled MCI due to AD and early AD dementia patients. It did not meet its primary endpoint (a composite score). A reduction in amyloid was observed at 18 months, with a low rate of ARIA-E incidence of less than 10%. Slowing of the cognitive composite was observed, and there was an increase in CSF Aβ42/40 during ttreatment^35^.

There is an ongoing phase III study (Clarity AD) for early symptomatic AD; and another study in the pre-clinical stages of AD (AHEAD3-45) is currently enrolling^36^.

### Gantenerumab

Gantenerumab is a monoclonal antibody with preferential affinity for Aß oligomers and fibril; it did not significantly affect biomarkers; mainly it did not reduce amyloid retention on amyloid PET. It reduced CSF p181-tau, but no differences were observed for ADAS-Cog, MMSE, and CDR-SB^37^. There is an ongoing phase III study with higher doses in MCI patients^37^. 

### Crenezumab

Crenezumab is a humanized anti- Aβ IgG4 mAb that recognizes the mid-domains of oligomeric and aggregated fibrillary forms of Aβ peptides and amyloid plaques. The two-phase two trials ABBY and BLAZE assessed safety, efficacy, amyloid burden change, and clinical outcomes in mild to moderate AD. The crenezumab group did not differ from the placebo group in both trials^38^ ^,^ ^39^. 

 Two phase-three trials, CREAD 1 and 2, designed to evaluate 1,500 patients with prodromal to mild AD in a two-year intervention, were discontinued in 2019 because of futility analysis^40^. The Alzheimer´s Preventiion Initiative (API) - Autosomal dominant Alzheimer disease (API-ADAD) trial is a double-blind placebo-controlled trial with crenezumab for patients with presenilin 1 mutation carriers^41^.

### Solanezumab

Solanezumab is a humanized immunoglobulin G1 monoclonal antibody that binds to the mid-domain of the Aβ peptide; its supposed action is to increase clearance of soluble Aβ from the brain^42^. 

 Solanezumab phase 1 and 2 studies showed that the drug is well tolerated with no serious adverse events. No inflammation, vasogenic edema, or microhemorrhage were demonstrated on MRI. The phase 2 study confirmed dose-dependent increases of various Aβ species in plasma and CSF but no clinical benefit^43^ ^,^ ^44^.

 Phase 3 trials, EXPEDITION-1 and -2, randomized 1,012 and 1,040 patients with mild to moderate AD, to receive 400 mg solanezumab or placebo monthly infusions for 18 months. Tolerability and safety have been confirmed, and no improvement on the primary outcome measures (ADAS-Cog11 and ADCS-ADL) was observed. Of note, in the two trials, approximately 25% of mild AD patients did not have evidence of amyloid-related disease^43^ ^,^ ^44^. 

 EXPEDITION-3, a phase 3 trial, enrolled 2,100 patients with mild AD with confirmed brain amyloid burden to receive 400 mg solanezumab or placebo monthly infusions for 76 weeks. The primary endpoint was ADAS-Cog 14. No clinical benefit was observed between treatment groups^45^.

 Solanezumab and gantenerumab were administered to asymptomatic and very mildly symptomatic carriers of autosomal-dominant mutations in the Alzheimer's genes APP, presenilin-1, and presenilin-2 for four to seven years. The trial failed to demonstrate cognitive benefits. An ongoing trial called A4 is a secondary prevention trial in asymptomatic or very mildly symptomatic people aged 65 and older who have biomarker evidence of brain amyloid deposition; this study will be completed in December, 2022 (clinicaltrials.gov; NCT02008357)^46^.

### Donanemab

Donanemab is a humanized immunoglobulin G1 monoclonal antibody that binds to a pyroglutamate form of Aβ present only in brain amyloid plaques, removing them from the brain of AD patients. A two-phase 1 clinical trial in patients with MCI or mild and moderate AD dementia showed a reduction in amyloid load, and safety and tolerability were demonstrated. Anti-drug antibodies were observed in most of the patients^47^ ^,^ ^48^.

 The phase 2 TRAILBLAZER-ALZ trial evaluated clinical benefits in a double-blind placebo-controlled trial in early or mild AD patients. Patients must have amyloid and tau pathology confirmed with flortaucipir and florbetapir PET scan, and received 700 mg for the first three doses and 1,400 mg intravenously every four weeks up to 72 weeks. Subjects with extensive tau pathology were excluded in order to avoid patients with advanced AD. Two hundred and seventy-two subjects were randomized, and the primary outcome was change from baseline to 76 weeks in Integrated Alzheimer’s Disease Rating Scale score, a combination of ADASCog13 and the Alzheimer’s Disease Cooperative Study-Instrumental Activities (ADCS-iADL), that ranges from 0 to 144. Secondary outcomes were: MMSE, Clinical Dementia Rating Scale Sum of Boxes (CDR-SB), and ADAS-Cog13 and ADCS-iADL separately. The Donanemab group declined by 6.86 points compared to 10.06 in the placebo group (p=0.04); baseline score was 106. Unfortunately, this difference did not reach the 6-point difference designated by the trial as a clinically relevant goal. Secondary outcomes did not differ between groups. Amyloid burden decreased in the donanemab group and remained stable in the placebo group. Tau pathology and hippocampal volume assessed by MRI did not differ between groups, but whole-brain volume had a greater decrease, while ventricular volume had a greater increase in the donanemab group. ARIA occurred in 38.9% of the participants in the donanemab group and 8.0% in the placebo group. ARIA-E was more frequent in the donanemab group than in the placebo group (26.7% vs. 0.8%) and was mostly asymptomatic. A phase 3 trial (TRAILBLAZER-ALZ 3) and a head-to-head trial with donanemab and aducanumab (TRAILBLAZER-ALZ 4) are in progress. (clinicaltrials.gov; NCT05026866; NCT05108922)^47^ ^,^ ^48^.

*6.6.1 No, we are not ready for anti-amyloid therapy in AD*

Several drugs have been tested in the past 20 years based on the amyloid cascade hypothesis. According to this theory, drugs that target the accumulation of amyloid would interrupt the pathogenic process of AD. The amyloid cascade could be stopped by decreasing Ab production, increasing brain Ab clearance, or antagonizing Ab aggregation. BACE inhibitors, passive immunotherapy, active immunotherapy, g-secretase inhibitors, g-secretase modulators, Ab aggregation inhibitors, and secretase inhibitors have all been tested, and most drugs failed in phase 1 or phase 2 trials^49^. Unfortunately, most drugs showed no significant clinical benefits, despite reducing brain amyloid burden, and many trials were halted for futility..

 The amyloid burden does not correlate with clinical severity of AD; cognitively healthy individuals may have amyloid deposition in their brains, and tau pathology has been observed prior to severe Ab deposition in young patients with AD^49^ ^,^ ^50^. Patients with suspected non-AD pathology (SNAP) have the same MCI-dementia conversion rate as individuals with Ab accumulation^50^. 

 Therefore, the central and starting pathology role of Ab accumulation in AD must be reviewed. It is reasonable to imagine that Ab deposition is a secondary consequence of another initial insult, or a brain reaction to neuronal damage^50^. Also, results of trials with genetically determined AD will be interesting to clarify the role of anti-amyloid therapy in these subgroups of patients.

 Even though we consider the ambiguous clinical benefits shown in some more recent monoclonal antibodies trials, we are still not prepared for this treatment: the high prevalence of AD worldwide demands an accessible treatment for many patients. This is not the case of monoclonal antibodies. For example, the estimated cost of aducanumab is US$56.000 yearly^51^. This cost does not consider cost of serial MRI or PET scans needed to evaluate drug response or the common side effect of ARIA. 

 In 2022, the American Academy of Neurology launched a position statement about the controversial approval of aducanumab by the FDA for the treatment of AD. In the statement, the ethical principles of beneficence and nonmaleficence are recalled to guide neurologists in the decision to prescribe the drug^52^. Until now data regarding the use of monoclonal antibodies do not prove clinical benefits, and the high potential for harm, mainly ARIA, suggest that anti-amyloid therapy is not ready for general use in AD.

*6.6.2 Yes, we are ready to prescribe and follow these patients in specialized dementia centers*

Favorable points for monoclonal antibodies use:

- Aducanumab is the first of a new era in AD treatment, a window of opportunity to understand the disease and to offer a therapy for disease modification;
- Infusion centers will be an adequate place to offer the treatment;
- A greater collaboration among primary care clinicians and specialists;
- There are observations of slowing of cognitive decline in other trials of antibodies, with an opportunity of hope for patients and caregivers.
